# Meningococcal Outbreaks in Tertiary Education Settings in the United Kingdom: Lessons from the 2026 Kent Cluster for Surveillance, Vaccination Policy, and Institutional Preparedness in Sub-Saharan Africa—A Narrative Review

**DOI:** 10.3390/idr18030051

**Published:** 2026-05-26

**Authors:** Malizgani Mhango, Enos Moyo, Nigel Tungwarara, Knowledge Denhere, Moses Chirimbana, Tafadzwa Dzinamarira

**Affiliations:** 1 The National Centre for Epidemiology and Population Health, College of Law, Governance and Policy, The Australian National University, Canberra, ACT 2601, Australia; 2 Africa Centre for Inclusive Health Management, Faculty of Economic and Management Sciences, Stellenbosch University, Stellenbosch 7602, South Africa; 3 Department of Health Studies, University of South Africa, Pretoria 0003, South Africa; nigelltung@gmail.com; 4 School of Public Health, University of Western Cape, Cape Town 7530, South Africa; kdenhere@rotunda.ie; 5 Department of Mechanical and Metallurgical Engineering, Faculty of Engineering and Information and Technology, University of Namibia, Ongwediva 15006, Namibia; 6 ICAP, Columbia University, Lusaka 10101, Zambia; 7 School of Health Systems and Public Health, University of Pretoria, Pretoria 0110, South Africa

**Keywords:** *Neisseria meningitidis*, sub-Saharan Africa, university outbreak, meningitis belt, MenAfriVac, serogroup B, campus preparedness, young adults, institutional health policy

## Abstract

Background: In March 2026, a meningococcal cluster centred on the University of Kent, England, caused two deaths and resulted in over 20 reported cases within the first week, including confirmed and suspected invasive cases. Subsequent UKHSA updates in early April 2026 reported 21 laboratory-confirmed MenB cases (18 linked to the outbreak strain) and two deaths, with the outbreak subsequently spreading to a second Canterbury university, Canterbury Christ Church University, and confirmed as Neisseria meningitidis serogroup B (MenB). Sub-Saharan Africa (SSA) bears a disproportionate global burden of meningococcal disease, yet university settings remain a critically understudied outbreak amplifier. This narrative review extracts epidemiological and policy lessons from the Kent event and applies them to the SSA context. Methods: We conducted a narrative review following the SANRA criteria, searching PubMed, Embase, Scopus, Google Scholar, and African Journals Online (2000–2026), with supplementary grey literature retrieved from World Health Organisation (WHO), Africa Centre for Disease Control, and United Kingdom Health Security Agency (UKHSA). Outbreak data were drawn from official UKHSA public-health statements (grey literature, archived), the University of Kent communications, and peer-reviewed expert commentary. Results: The Canterbury outbreak exposed six reproducible vulnerabilities: unprotected serogroup circulation (confirmed MenB, not covered for the current university-age cohort), nightlife-linked transmission amplification, delayed serogroup identification, poor student symptom-recognition, inadequate institutional response capacity, and, critically, multi-institutional spread via shared nightlife venues (confirmed extension to Canterbury Christ Church University within five days). Each vulnerability is demonstrably more severe in SSA universities, which face a broader multi-serogroup threat environment (NmA, B, C, W, X), virtually no university-entry vaccination requirement, and critical evidence gap of campus-specific meningococcal evidence in the published literature. Conclusions: This review proposes a five-pillar preparedness framework for SSA tertiary institutions, derived from a synthesis of the Kent outbreak and broader epidemiological evidence, intended to inform policy discussion and future research. Moreover, these should be embedded within a broader age-linked prevention strategy that begins before university entry, particularly during the transition into secondary school in high-risk settings. Priority measures include meningococcal vaccination at key educational transition points, prophylactic antibiotic pre-positioning, serogroup-capable surveillance, symptom-recognition training, and pan-continental alert *A* predominantly reactive response may carry substantial risk in SSA settings.

## 1. Introduction

Meningococcal disease, caused by the encapsulated Gram-negative bacterium *Neisseria meningitidis*, is among the most feared infections in human medicine. It combines unpredictable onset with catastrophic progression, carrying a case fatality rate of 10–15% even with optimal treatment in high-income settings; rates in sub-Saharan Africa (SSA) without immediate antibiotic access exceed 20–30% [1,2]. Its capacity to kill a healthy young adult within 24 h of symptom onset, and its efficient spread through respiratory droplets in confined indoor spaces, renders the modern tertiary education campus one of the most epidemiologically volatile environments for meningococcal transmission globally [3].

On 13 March 2026, the United Kingdom (UK) Health Security Agency (UKHSA) was notified of the first confirmed cases of invasive meningococcal disease (IMD) at the University of Kent, Canterbury, England [4]. By 15 March—72 h later—13 cases combining meningitis with septicaemia had been confirmed, two individuals had died (a 21-year-old University of Kent student and an 18-year-old pupil at Queen Elizabeth’s Grammar School, Faversham), and epidemiological investigation had linked multiple cases to a social gathering at a nightclub in Canterbury between 5 and 7 March 2026, a link subsequently confirmed by UKHSA investigation [4,5,6]. The UKHSA mobilised a public health emergency response, alerting over 30,000 individuals, distributing over 6500 prophylactic antibiotic doses across campus and community sites, coordinating academic suspension for the affected week, and initiating a targeted Meningococcal group B (MenB) vaccination programme for approximately 5000 University of Kent students’ resident on the Canterbury campus [6,7]. By 17–18 March, four to five days after the first notification, the causative serogroup was confirmed as *Neisseria meningitidis* group B (MenB), with six cases laboratory-confirmed and 14 further cases under investigation; the total number of reported cases had risen to 20 (including confirmed and suspected cases) by 18 March 2026. Subsequent UKHSA updates in early April 2026 confirmed 21 laboratory-confirmed MenB cases, of which 18 were identified as part of the outbreak strain subtype P1.12-1,16-183, with 2 deaths, and the outbreak had spread to a second Canterbury institution, Canterbury Christ Church University (CCCU), illustrating that even within the first week of an intensified response, institutional containment was incomplete [7].

Sub-Saharan Africa (SSA) bears an incomparably greater burden of meningococcal disease. The African meningitis belt, spanning 26 countries from Senegal to Ethiopia, has historically sustained attack rates reaching 1000 cases per 100,000 at the district level during peak epidemic seasons [8]. The mass deployment of the Meningitis Africa Vaccine (MenAfriVac) from 2010 produced one of Africa’s greatest public health achievements, essentially eliminating serogroup A (NmA) epidemics [9,10]. Yet this success has driven a critical transition: the emergence of non-*Neisseria meningitidis* serogroup A (NmA) serogroups—*Neisseria meningitidis* serogroup W (NmW), *Neisseria meningitidis* serogroup C (NmC), *Neisseria meningitidis* serogroup X (NmX), and *Neisseria meningitidis* serogroup B (NmB)—for which continent-wide vaccine coverage remains severely inadequate [11,12]. Simultaneously, SSA tertiary education institution enrolment has nearly tripled over two decades, from approximately 3.5 million to over 10 million enrolled students by 2022 [13], creating densely populated campuses that are ideal meningococcal amplifiers yet lack systematic outbreak preparedness [14,15,16].

This narrative review pursues four aims: (i) to document the epidemiology and public health response of the 2026 Kent outbreak; (ii) to synthesise evidence on meningococcal burden, serogroup ecology, and vaccination policy in SSA; (iii) to examine the meningococcal risk landscape in SSA tertiary education institutions; and (iv) to propose a GRADE-informed, preparedness-oriented framework of institutional policy options for tertiary settings. This review is intended as a hypothesis-generating and policy-informing synthesis, rather than a definitive evidence-based guideline, reflecting the current absence of direct SSA campus-specific meningococcal data.

## 2. Methods

### 2.1. Study Design and Reporting Framework

This paper is a narrative review, and the methodological designation is applied consistently throughout this manuscript in accordance with the Scale for the Assessment of Narrative Review Articles (SANRA) reporting framework [17]. Narrative review was selected because the evidence base spans heterogeneous study designs, epidemiological surveillance data, clinical case series, outbreak investigations, health policy documents, and vaccination programme evaluations, and the urgent policy context created by an active outbreak event required synthesis rather than strictly systematic methods. The manuscript conforms to all six SANRA assessment criteria: (i) justification of the review’s importance; (ii) statement of aims and literature search; (iii) appropriate use of a database search; (iv) citation of appropriate literature; (v) scientific reasoning; and (vi) appropriate presentation of data. A SANRA compliance checklist is provided as Supplementary File S1.

### 2.2. Literature Search

PubMed/MEDLINE, Embase, Scopus, Google Scholar, and African Journals Online (AJOL) were searched for peer-reviewed literature published between January 2000 and March 2026. For AJOL, additional search terms were used, including ‘cerebrospinal meningitis,’ ‘bacterial meningitis students,’ and ‘meningococcal Africa university’ alongside standard English MeSH terms. Searches were conducted independently by two reviewers (MM and EM), with discrepancies resolved by consensus; no formal inter-rater reliability statistic was computed, consistent with the narrative review methodology [17]. Keyword combinations included: *Neisseria meningitidis* OR meningococcal disease OR invasive meningococcal disease AND sub-Saharan Africa, Africa; meningitis belt AND epidemiology; university OR college OR campus OR tertiary students AND meningococcal AND outbreak; MenAfriVac AND serogroup; meningococcal vaccine AND young adults AND Africa; outbreak preparedness AND higher education. Reference lists of all included articles were hand-searched. The search start date of 2000 was selected as the period from which contemporary serogroup epidemiology in the post-genomic era is reliably documented; seminal pre-2000 papers are cited for historical context where necessary.

Grey literature was retrieved from the World Health Organisation (WHO), Africa Centre for Disease Control (CDC), UKHSA, the CDC, and the Gavi Alliance. The 2026 Canterbury outbreak data were drawn from UKHSA public health statements (15–19 March 2026; archived versions at web.archive.org), the UKHSA Clinical Alert System (CAS) advisory issued to National Health Services (NHS) England on 17 March 2026, the NHS England outbreak briefing note (17 March 2026), University of Kent official communications (archived), Canterbury Christ Church University official communications (18 March 2026; archived), and expert commentary published by the Science Media Centre. These sources are identified throughout with their limitations formally addressed in Section 9.5.

Data were extracted independently by two reviewers (MM and EM) using a standardised extraction form capturing: study design, setting, country, population, sample size, serogroup(s) investigated, key outcomes relevant to meningococcal epidemiology or campus risk, and principal study limitations. For the grey literature and outbreak investigation reports, the extraction captured case counts, event dates, interventions deployed, and source quality indicators. No formal meta-analytic synthesis was performed, given the substantial heterogeneity across included study designs; findings are presented as a narrative synthesis organised thematically by section. Discrepancies between reviewers during extraction were resolved by consensus discussion; no formal inter-rater reliability statistic was calculated, consistent with narrative review methodology. The reliance on grey literature for the Kent outbreak reflects the recency of the event; such sources were critically appraised and used primarily for descriptive epidemiology rather than inferential claims.

### 2.3. Inclusion Criteria and Evidentiary Hierarchy

Studies were included if they reported on meningococcal disease epidemiology, serogroup dynamics, vaccine policy, or outbreak response in SSA; or on meningococcal disease in university or comparable institutional settings globally; or on young adult (18–25 years) meningococcal risk. Studies were excluded if published before 2000 (except foundational papers), unavailable in English, or restricted to non-meningococcal meningitis without comparative data.

Given the near-complete absence of SSA higher education institution (HEI)-specific meningococcal data—itself a key finding of this review—the evidentiary architecture was organised into three tiers: (Tier 1) direct SSA HEI evidence, which is almost entirely absent; (Tier 2) SSA general-population meningococcal epidemiology, extrapolated to the HEI context with acknowledged limitations; and (Tier 3) high-income-country HEI meningococcal evidence, applied by analogy. Conclusions and recommendations are weighted accordingly, and extrapolation from HIC settings is explicitly qualified throughout, acknowledging potential confounders including different predominant serogroups; different population immunity profiles from prior MenAfriVac exposure; different co-infection profiles (HIV, sickle cell disease); and different healthcare-seeking behaviours. The single available SSA HEI-specific study employed a cross-sectional, self-report design. Data extraction included study design, participant characteristics, exposure measures, and outcome definitions. Study quality was appraised using the STROBE checklist [18], which identified key limitations, such as self-report bias and potential selection bias. Accordingly, the findings are interpreted as hypothesis-generating rather than confirmatory.

### 2.4. Evidence Grading for Recommendations

Recommendations in Section 8 are presented using a GRADE-informed heuristic framework, adapted to distinguish between quality of evidence and strength of recommendation in a context where direct SSA-specific data are limited [19] “Strong” recommendations indicate that desirable effects clearly outweigh undesirable effects based on available evidence; “Conditional” recommendations indicate that benefits are expected, but uncertainty about evidence quality or implementation context warrants flexibility. Quality of Evidence reflects the extent to which available studies directly support the recommendation in the SSA HEI context.

Duplicate sources identified during the review process were merged under a single citation number throughout. Deduplication was performed using Rayyan (Qatar Computing Research Institute; available at https://www.rayyan.ai/?_ga=2.84812378.21207942.1773887842-956696301.1773887842 (accessed on 19 March 2026) during title and abstract screening. This approach is intended to provide structured transparency rather than to imply full formal GRADE applicability.

### 2.5. Use of Artificial Intelligence Tools

The authors acknowledge the use of Claude (version 3.5 Sonnet; Anthropic PBC; San Francisco, CA, USA; https://www.anthropic.com/claude, accessed on 19 March 2026) and ANSWERTHIS (AnswerThis Ltd.; London, UK; https://app.answerthis.io/ask-answerthis?style=full-review, accessed on 19 March 2026) during the preparation of this manuscript. Claude was used for language refinement, academic tone improvement, and methodological structuring, while ANSWERTHIS was used to support literature search and mapping. All outputs generated with the assistance of these AI tools were critically reviewed, edited, and validated by the authors. No AI tools were used for independent data interpretation or for generating scientific conclusions. The authors confirm full responsibility for all content presented in this manuscript.

## 3. The 2026 University of Kent, England Meningococcal Outbreak

### 3.1. Institutional Context and Timeline

The University of Kent, Canterbury in England, hosts approximately 20,000 enrolled students across its residential campus [6]. Canterbury is also home to several grammar schools whose students share social spaces with university students, a feature with direct epidemiological significance in the 2026 cluster, which claimed a non-university fatality. Epidemiological reconstruction identified a nightclub in Canterbury as the primary exposure venue on 5–7 March 2026; this link was subsequently confirmed by UKHSA investigation, with at least 10 of 20 cases having attended the venue on those dates [4,5]. Reported case counts during the early phase included both confirmed and suspected cases and are presented as such where relevant. Between 13 and 18 March 2026, 20 reported cases of invasive meningococcal disease were notified to UKHSA—one of the largest and most rapidly evolving university meningococcal clusters in recent UK history, described by the UK Health Secretary as “unprecedented” in pace and extent [4]. Two people died within this 72 h window. By 15–17 March, UKHSA had alerted over 30,000 individuals, established multi-site antibiotic distribution, suspended in-person assessments, and extended the prophylaxis offer to all attendees to the nightclub in question from the relevant dates [6,7]. As of 17–18 March, six cases had been laboratory-confirmed as *Neisseria meningitidis* group B (MenB); the remaining 14 cases were under investigation. This four-to-five-day interval between first notification and serogroup confirmation in one of the world’s most capable public health reference systems illustrates the structural diagnostic bottleneck that even high-income systems face, with profound implications for SSA. Furthermore, by 18th March, the outbreak had extended beyond the University of Kent to Canterbury Christ Church University (CCCU), where one confirmed case was identified in a student who had also attended the Club during the exposure window [7]. At the time of initial manuscript preparation (17–19 March 2026), the outbreak was still evolving, and case counts included both confirmed and suspected cases. Subsequent UKHSA updates in early April 2026 clarified the outbreak trajectory, confirming 21 laboratory-confirmed MenB cases (18 outbreak strain) and 2 deaths. These updates reinforce, rather than alter, the interpretation of the outbreak as a rapidly progressing, high-risk campus cluster.

### 3.2. Five Epidemiological Features of Relevance to SSA

#### 3.2.1. Velocity of Cluster Progression

Twenty confirmed and suspected invasive cases within six days, with 13 in the first 72 h, is consistent with hyper-acute cluster dynamics driven by a single high-density social exposure event, in this case confirmed as a nightclub in Canterbury. The subsequent spread to CCCU further illustrates that in a shared urban nightlife environment, institutional boundaries may not provide a reliable epidemiological firebreak in settings where transmission is driven by shared social environments rather than formal institutional contact networks: a single venue served as the common exposure nexus across two HEIs, at least one grammar school, and the wider Canterbury community within days. In SSA, where surveillance and response systems operate more slowly, the interval between exposure and effective containment would almost certainly permit additional transmission cycles [20].

#### 3.2.2. Nightlife as Transmission Amplifier

Indoor nightlife venues create near-perfect conditions for respiratory-pathogen transmission: prolonged occupancy, elevated vocal volume, close physical proximity, and alcohol-related disinhibition. UK case–control studies have identified nightclub attendance as a statistically significant and independent risk factor for meningococcal serogroups C and W, with adjusted odds ratios of 3.1–4.7 compared with non-attendees after adjustment for other social behaviours [21,22]. Smoking, documented as an independent meningococcal risk factor in case–control studies [23], is co-prevalent in nightlife settings, further increasing transmission risk.

#### 3.2.3. Delayed Serogroup Identification

Serogroup identification is not a taxonomic exercise—it directly determines which vaccine can be offered as post-exposure prophylaxis (PEP) supplementary to antibiotics [24]. The MenACWY vaccine offered to UK teenagers does not protect against NmB, the serogroup responsible for most UK university deaths in the preceding two decades [25]. Critically, the UK infant NmB programme (4CMenB, introduced 2015) does not cover the current university-age cohort: students aged 18–24 in 2026 were born 1998–2007, predating universal infant NmB vaccination. In the Kent 2026 outbreak, six cases were laboratory-confirmed as MenB by 17–18 March—a four-to-five-day interval from first notification to serogroup confirmation in one of the world’s best-equipped reference systems. This delay underscores how much more severe this bottleneck will be in SSA, where the equivalent confirmation period is measured in days to weeks [26,27].

The structural roots of this bottleneck in SSA are well-documented. A global systematic review and survey of meningococcal surveillance found that approximately 30% of countries either relied exclusively on national reference laboratories for serogrouping or did not routinely serogroup specimens at all, with SSA countries disproportionately concentrated in this group [28]. In a landmark external quality assessment of 81 national public health laboratories from 45 SSA countries participating in the WHO/NICD programme, it was reported that while 76% of participating laboratories achieved acceptable scores for bacterial identification, only 42% achieved acceptable scores for antimicrobial susceptibility testing—the complementary investigation necessary to guide treatment choice [29]. Critically, these assessments enrolled laboratories specifically engaged in WHO capacity-building programmes; performance among non-enrolled district and campus-referral laboratories would be expected to be substantially lower. On quantifying the downstream surveillance consequence, it was found that more than 75% of reported meningococcal meningitis cases across belt countries lacked laboratory confirmation of the causative strain, with extreme inter-country heterogeneity—in 2016, only 22 of 831 (2.6%) suspected invasive meningococcal disease cases in Nigeria had laboratory-confirmed serogroup identification, compared to 86% in neighbouring Niger [11].

The infrastructure deficit underlying these figures is severe. Researchers identified only 380 laboratories across all of SSA accredited to international quality standards, with 91% concentrated in South Africa, and 37 of 49 SSA countries possessing no internationally accredited laboratory whatsoever [30]. The defining constraints were described as a triad of inadequately trained laboratory personnel, insufficient reagents and equipment, and the absence of functional quality management systems—constraints that have persisted across the region for decades and directly impede serogroup-level meningococcal diagnostics [31]. Polymerase Chain Reaction (PCR)-based serogroup typing—essential for NmX and NmB identification, neither of which is reliably detectable by standard latex agglutination tests—is confined to a small number of national reference laboratories, requires cold-chain sample transport (4 °C or −20 °C), and returns results in days to weeks, rendering confirmation non-actionable within the clinically critical response window [5,27]. In the HEI outbreak context, where serogroup identification determines which vaccine should be offered as PEP and which student cohorts require priority notification, this multi-layered diagnostic delay represents a critical structural vulnerability with no short-term solution, an absent deliberate and sustained infrastructure investment.

#### 3.2.4. Symptom Recognition Failure

UKHSA cautioned that students commonly misidentify early meningococcal symptoms, such as fever, headache, photophobia, and myalgia, as viral illness or hangover [7]. Research among university students confirms that fewer than 40% correctly identify the non-blanching rash as a cardinal danger sign, with knowledge lowest among male students, international students, and first-year entrants [27]—subgroups prevalent in SSA HEI populations [16]. In SSA settings, where adolescents in boarding schools may carry a comparable burden of early recognition, this training logic may reasonably extend beyond university staff to teachers, house-parents, and dormitory supervisors. This subgroup disparity has direct implications for targeted intervention design in SSA HEI settings.

#### 3.2.5. Response Benchmark and Its Gaps

The UKHSA’s response: same-day notification, multi-site antibiotics within 24 h, a 30,000-person alert within 48 h, and deployed six established preparedness components simultaneously [32]. Yet even this response left critical gaps: a four-to-five-day delay in serogroup confirmation (subsequently identified as MenB); incomplete nightclub attendance records impeding contact tracing; a secondary-school fatality demonstrating community transmission beyond the university network; and, critically, institutional spread to a second university—CCCU—confirming that campus boundaries provide no epidemiological barrier within a shared nightlife environment. The CCCU case, confirmed on 18 March 2026 and directly linked to the nightclub, represents structural proof-of-concept: a single high-density social venue served as the common exposure nexus across two separate higher education institutions, a grammar school, and the wider Canterbury community simultaneously. These compound failures in a well-resourced system serve as a direct and sobering warning for SSA, where no equivalent preparedness infrastructure exists [20].

#### 3.2.6. Spread to a Second University: Canterbury Christ Church University

On 18 March 2026, CCCU confirmed that one of its students had been diagnosed with meningococcal disease, directly linked to attendance at Club Chemistry during the 5–7 March exposure window [33,34,35]. CCCU Vice-Chancellor confirmed the case publicly, stating that the institution had informed close contacts and advised precautionary antibiotics in accordance with UKHSA guidance. The CCCU campus remained open, with core teaching and research activities continuing. This extension beyond the University of Kent likely reflects a shared exposure environment; however, it also illustrates how a single high-density social venue can link multiple institutions within a short transmission window, a dynamic that may be particularly relevant in SSA university cities. First, it demonstrates that a single shared nightlife venue can serve as a cross-institutional transmission hub, simultaneously seeding cases across multiple higher education institutions without any direct university-to-university contact. In SSA HEI cities, Accra, Lagos, Nairobi, Dar es Salaam, Kampala, Harare, and Johannesburg, multiple HEIs routinely share commercial nightlife, transport hubs, and social spaces, creating equivalent or greater cross-institutional exposure networks. Second, it confirms that single-institution containment protocols are structurally insufficient in the absence of coordinated multi-institution outbreak management: prophylaxis offered only to University of Kent students cannot reach CCCU students sharing the same exposure event at the same venue. Third, the rapid identification of the CCCU case, within five days of the initial cluster notification, illustrates both the relative efficiency of UKHSA contact tracing and, by contrast, the near-certain absence of any comparable cross-institutional surveillance mechanism in SSA. In the SSA context, a CCCU-equivalent case would most likely be clinically managed as an isolated community presentation, with the link to a cluster at a neighbouring university either never established or identified only after additional secondary cases had occurred.

## 4. Meningococcal Disease in Sub-Saharan Africa: Burden, Serogroup Ecology, and Surveillance

### 4.1. Historical Burden and the Meningitis Belt

The African meningitis belt was first delineated by Lapeysonnie in 1963, describing the geographic regularity of seasonal meningococcal epidemics across a semi-arid band spanning 26 countries [36]. Before the MenAfriVac era, epidemic years produced 30,000–200,000 reported cases across the belt, with attack rates reaching 1000 per 100,000 at the district level during the dry harmattan season [8,37]. Hot, desiccating harmattan conditions compromise nasopharyngeal mucosal defences [38,39], and the periodic introduction of hypervirulent clonal lineages into partially immune populations drives explosive epidemic curves with case doubling times of three to five days [40]. Long-term sequelae affect approximately 10–15% of survivors, including sensorineural deafness and cognitive impairment [41].

### 4.2. MenAfriVac: Achievement and Post-Introduction Epidemiological Transition

The Meningitis Vaccine Project (MVP) developed MenAfriVac as a low-cost, heat-stable, monovalent NmA conjugate vaccine for the meningitis belt [42]. Mass campaigns beginning in December 2010 vaccinated over 220 million persons aged 1–29 years, representing the catch-up campaign target range, across 16 belt countries by 2015, before transitioning into routine infant expanded programme on immunisation (EPI) schedules [9,42]. This distinction matters: current SSA HEI students (likely born 1998–2007) fall within the catch-up campaign age range and were eligible for MenAfriVac during the 2010–2015 rollout. However, campaign coverage was geographically heterogeneous and did not reach all individuals in this cohort. Researchers documented a 99.8% reduction in NmA incidence in vaccinated cohorts across nine belt countries between 2010 and 2015 [9]. NmA epidemic meningitis has been essentially eliminated from vaccinated populations, one of the most dramatic vaccine-attributable disease reductions achieved in any low-income setting.

However, this success has revealed a critical multi-serogroup threat landscape in which non-NmA serogroups—previously overshadowed by NmA’s epidemic dominance—now predominate [11].

### 4.3. The Multi-Serogroup Threat: NmW, NmC, NmX, and NmB

Serogroup W (NmW), driven by the hypervirulent Sequence Type (ST)-11 clonal complex introduced via worshippers attending the annual Muslim Hajj pilgrimage in Mecca, Saudi Arabia, in 2000 [43], has caused major outbreaks across Burkina Faso, Ghana, Togo, and South Africa, displacing NmB as the dominant serogroup from 2015 onwards [44,45]. Its clinical predilection for septicaemia over meningitis is associated with more rapid deterioration and higher pre-hospital mortality [43]. Serogroup C (NmC) re-emerged in Nigeria and Niger’s 2013–2015 wave and again in Niger’s 2022–2023 Zinder outbreak, which documented 559 cases (93.7% NmC) and 18 deaths within a single season, with confirmed cross-border spread to Nigeria’s Jigawa State [46,47,48]. Serogroup X (NmX) has caused outbreaks in at least six belt countries and poses a unique dual challenge: it is covered by no currently licensed meningococcal vaccine, and standard peripheral rapid diagnostic tests cannot reliably detect it, creating a surveillance blind spot [27,49]. A pentavalent MenACWYX conjugate vaccine, currently in late-stage development, has demonstrated high immunogenicity across all five serogroups in phase II/III trials. As of early 2026, it had not yet achieved full WHO pre-qualification or widespread regulatory approval, and its licensure status for routine use in older adolescents and young adults (including the 18–25 age group) remains in transition pending regulatory review and programmatic adoption.; programmatic rollout into SSA national schedules, including for university-entry cohorts, remains incomplete [50]. Serogroup B (NmB) predominates in North Africa, South Africa, and in university outbreaks globally [51,52]. NmB vaccines (4CMenB; rLP2086), based on protein subcapsular antigens, are immunogenic and effective, but are priced at USD 25–80 per dose in SSA private markets, inaccessible for routine national programmes—leaving SSA HEI students unprotected against the serogroup most likely to cause campus clusters [53,54].

### 4.4. Surveillance Architecture and Limitations

Meningococcal surveillance in SSA operates through the WHO Integrated Disease Surveillance and Response (IDSR) framework, with epidemic thresholds of 10–15 cases per 100,000 per week calibrated primarily to NmA epidemic detection at the district level [55]. These thresholds may be insensitive to localised HEI clusters in the post-MenAfriVac era; researchers documented persistent underreporting across belt-country surveillance systems [37]. Updated surveillance thresholds appropriate to the multi-serogroup era are under active WHO review [56]. The absence of routine whole-genome sequencing or multilocus sequence typing (MLST) at most sub-Saharan Africa national reference laboratories means that serogroup confirmation, essential for reactive vaccine selection, can take days to weeks, compared to same-day confirmation in UKHSA’s reference system, which itself failed to characterise the Kent strain within four days [27].

These systemic diagnostic limitations are compounded by documented shortfalls in human resources for laboratory health. Meningitis surveillance across SSA remains characterised by parallel, non-synergistic reporting systems and critically limited laboratory capacity, conditions that collectively undermine the timely outbreak detection on which any effective campus response depends [57]. Where PCR-based serogrouping is available at the national reference level, specimen transport chains are unreliable, cold-chain failures are common, and reagent stockouts are documented even in purpose-built WHO/MenAfriNet surveillance networks [11,58]. The shortage of trained laboratory scientists with competence in bacterial culture and serogroup identification is a continent-wide structural problem, with the lack of adequately skilled laboratory personnel as the primary limiting factor for diagnostic capacity in SSA, ahead of equipment shortages and funding gaps [59]. Critically, only 380 laboratories across all 49 SSA countries met international quality standards as of the most recent comprehensive survey, 91% of which were located in South Africa, meaning that the vast majority of SSA countries operate without a single internationally accredited laboratory to support outbreak diagnosis [60]. Taken together, these limitations ensure that the epidemiological information essential for reactive vaccine selection in a campus outbreak will arrive either too late or not at all—a structural reality that the Kent four-day identification delay, itself occurring in one of the world’s best-equipped surveillance systems, barely begins to approximate.

One practical implication is the need for decentralised rapid diagnostic capacity during suspected campus clusters. Where validated assays are available and nationally approved, ministries of health and university-linked referral systems should prioritise procurement pathways for near-patient or field-deployable diagnostic tools that can accelerate presumptive meningococcal identification and shorten the interval to appropriate public-health action. These tools will not eliminate the need for confirmatory reference-laboratory testing, but they may reduce the operational delay that currently renders serogroup information non-actionable during the most critical early phase of outbreak response.

## 5. The University Amplifier: Meningococcal Risk in SSA Tertiary Institutions

### 5.1. Global Evidence on HEI Campus Risk

The meningococcal risk in young adults concentrated on HEI campuses is one of the best-characterised phenomena in infectious disease epidemiology. The fundamental mechanism, a non-immune host encountering novel strains carried by geographically diverse peers in a dense social and residential environment, was first formally documented in the United States [61]. Rosenstein et al. established that college-age students, particularly those residing in dormitories, faced significantly elevated meningococcal disease incidence compared to age-matched non-students [62], a finding confirmed by Mbaeyi et al., who documented an 11.8-fold higher serogroup B incidence among first-year undergraduates compared to non-student peers aged 18–24 [14]. Soeters et al. described 10 US university NmB outbreaks between 2013 and 2018—39 cases, two deaths, outbreak durations up to 376 days—illustrating the protracted nature of campus meningococcal events within dynamic student social networks [63]. Parikh et al.’s data underpinned the UK’s 2015 decision to introduce the infant NmB programme, in part by documenting the excess campus NmB risk among university first-year students [64]. Alcohol consumption, nightclub attendance, and active smoking are independent risk factors documented across multiple settings [21,22,23].

### 5.2. Meningococcal Carriage Dynamics

Meningococcal carriage—asymptomatic nasopharyngeal colonisation, is the substrate from which all invasive disease arises, with the probability of any colonisation event producing invasive disease estimated at 1 in 1000 to 1 in 100,000 [3]. Campus environments dramatically accelerate carriage acquisition: Neal et al. documented an increase in pharyngeal meningococcal carriage prevalence from approximately 6% at university entry to over 23% by the end of the first year [24], while Imrey et al. demonstrated that dormitory-residing first-year students acquired new strains at a rate approximately 4.5 times that of non-dormitory peers, concentrated in the first three months of the academic year, the period of maximum freshers’ social intensity [65].

### 5.3. Evidence from SSA HEI Settings

A comprehensive search of PubMed, Embase, and AJOL identified a near-total lack of peer-reviewed primary research reporting meningococcal incidence, carriage prevalence, or outbreak investigations within SSA tertiary education institutions. The closest SSA-specific evidence comes from South Africa: a 2025 Public Health Association of South Africa (PHASA) policy brief, drawing on National Institute for Communicable Diseases (NICD) surveillance data, documented a pattern of recurrent meningococcal deaths in student residences, noting that barely a year passes without a residential case [16]. Young adults aged 15–24 years bear the second-highest age-specific meningococcal burden in South Africa, driven by the ‘new strain meeting naive host’ mechanism. Despite this, no SSA country had implemented mandatory university-entry meningococcal vaccination as of March 2026 [16]. One cross-sectional self-report study from Nigeria [66] found higher self-reported prior meningococcal illness among students attending social events more than twice weekly; however, this study carries substantial methodological limitations, self-report of provider-diagnosed prior illness, cross-sectional design, recall bias, and inability to establish causal inference, and its findings should be treated as hypothesis-generating rather than confirmatory.

### 5.4. Structural Risk Amplifiers in SSA HEI Campuses

Beyond the universal risk factors, SSA campus environments contain structural features that further amplify meningococcal transmission. Residential overcrowding is substantially higher than in higher-income countries (HIC) universities: room-sharing among 4 to 8 students is reported across public universities in Nigeria, Ghana, Kenya, Tanzania, Uganda, and Ethiopia [67]. Ventilation in older SSA residential buildings—many constructed in the 1960s–1970s—frequently fail to meet current public health standards. Geographic and immunological diversity among SSA students is even greater than in European universities: the rapid growth of inter-African student mobility schemes has produced campuses populated by students from diverse meningococcal exposure histories [13], increasing the probability of the ‘new strain meeting naive host’ mechanism described by Caugant and Maiden as the fundamental prerequisite for sporadic case and cluster generation [3]. Importantly, several of these structural risk amplifiers are not unique to universities. In many SSA countries, secondary boarding schools also combine residential crowding, prolonged indoor contact, geographically mixed student populations, and delayed access to definitive medical care. These features make them epidemiologically analogous to university dormitory environments for meningococcal transmission. Although the present review focuses on tertiary institutions, the preparedness logic described here is likely to be relevant to boarding secondary schools, particularly in meningitis-belt settings.

### 5.5. SSA-Specific Risk Modifiers: HIV, Sickle Cell Disease, and Gender

The meningococcal risk landscape in SSA HEI populations is shaped by three biologically important risk modifiers absent from European and North American campus contexts.

HIV infection increases the relative risk of invasive meningococcal disease approximately 10-fold through impaired complement-mediated opsonisation and reduced B-cell function [68]. HIV prevalence among university-age adults (18–25 years) in southern and eastern Africa ranges from 2% to over 15%, depending on country, gender, and rural-urban origin—far exceeding the HIC baseline. Meningococcal vaccination in HIV-positive individuals requires careful consideration of response efficacy, and routine vaccination protocols for immunocompromised students should be explicitly incorporated into any SSA HEI vaccination policy [68].

Sickle cell disease (SCD), which is highly prevalent across SSA, is associated with functional asplenia and markedly elevated susceptibility to invasive encapsulated bacteria, including *Neisseria meningitidis*. Vaccination recommendations for SCD patients typically include additional doses of all available meningococcal conjugate vaccines, reinforced antibiotic prophylaxis, and prioritised campus health monitoring—protocols that are absent from SSA HEI health services [69].

Male sex is an independent risk factor for meningococcal disease in HIC campus settings, with risk ratios of approximately 1.5–2.0, attributed to differences in nightlife attendance frequency, alcohol consumption patterns, symptom-reporting thresholds, and healthcare-seeking delay [21,22]. Gender-disaggregated approaches to symptom-recognition training and campus surveillance are therefore warranted in SSA HEI health policies.

## 6. Vaccination Policy: The Young Adult Gap and Path to Multi-Serogroup Protection

### 6.1. Current Vaccine Landscape in SSA

MenAfriVac remains the cornerstone meningococcal vaccine across the meningitis belt, primarily delivered through routine infant EPI schedules following transition from mass campaigns [9,42]. Polysaccharide ACWY vaccines are used reactively during confirmed outbreaks, providing short-term protection but lacking the immunological durability of conjugate vaccines and conferring no herd protection through carriage reduction [70]. Quadrivalent MenACWY conjugate vaccines, incorporated into UK and US adolescent programmes, are available in some SSA countries only through private providers at USD 25–80 per dose, inaccessible to the majority of students [71]. Gavi Alliance support for meningococcal vaccines in eligible SSA countries covers MenAfriVac and, prospectively, the MenACWYX pentavalent vaccine upon pre-qualification [19,71].

### 6.2. The Young Adult Vaccine Gap

SSA meningococcal immunisation strategies have historically targeted children aged 1–5 years and reactive mass vaccination of 1–29-year-old cohorts during outbreaks [8,42]. University students (18–25 years) occupy an epidemiologically critical but programmatically neglected zone: too old for infant schedules, too young to have accumulated substantial natural immunity, and socially positioned in the highest-risk campus environment. This ‘young adult vaccine gap’ is directly analogous to the vulnerability exposed by the Kent outbreak—where the unprotected serogroup was NmB rather than absence of vaccination entirely—but in SSA, the gap is wider (multiple serogroups) and deeper (no institutional vaccination requirement) [4,25]. In addition, the risk window may begin before university age. In high-burden settings, this raises the possibility that a time-limited catch-up strategy spanning older school-aged children, adolescents, and young adults (for example, approximately 10–24 years, adapted to national epidemiology and vaccine availability) could offer greater population benefit than a university-only approach. We present this not as a universal one-size-fits-all prescription, but as a programmatic option for countries seeking to close residual immunity gaps left by heterogeneous prior campaign coverage [4,25]. However, in the SSA context, university entry should not be interpreted as the first or only meaningful vaccination opportunity. Evidence from the African meningitis belt indicates that meningococcal carriage and disease risk can rise earlier in adolescence, particularly around 10–14 years of age. For this reason, university-entry vaccination is best understood as a late catch-up and risk-reduction measure within a broader age-linked strategy that should ideally begin at the transition from primary to secondary school, especially in meningitis-belt countries and in settings with boarding-school residence.

### 6.3. Vaccine Hesitancy

Effective university-entry vaccination programmes in SSA must contend with documented vaccine hesitancy among young African adults. A cross-sectional survey among Nigerian university students found that concerns about side effects, distrust of pharmaceutical companies, and COVID-19 vaccine-related misinformation were independent predictors of hesitancy towards recommended vaccines [72]. Historical colonial public health legacies, political instrumentalisation of health programmes in conflict-affected settings, and social media-driven misinformation further amplify hesitancy dynamics in SSA compared to HIC settings [29]. Co-designed, culturally grounded campus communication strategies developed in partnership with student unions are essential components of any vaccination mandate implementation.

Top-down risk communication alone may be insufficient in settings where institutional mistrust or misinformation is prominent. Peer-led and youth-facing communication models may therefore offer a more credible route to improving both vaccine uptake and early symptom recognition. In the SSA context, campus preparedness strategies should consider structured engagement with student associations, peer educators, and youth ambassadors so that meningococcal awareness is communicated through trusted social networks rather than only through administrative channels.

## 7. Institutional Outbreak Preparedness: From the UKHSA Benchmark to the SSA Reality

### 7.1. Six Components of Effective Meningococcal Response

Effective institutional meningococcal outbreak response requires simultaneous activation of: (i) rapid passive case notification; (ii) clinical management for severe invasive disease; (iii) rapid serogroup-typing capacity; (iv) contact tracing with defined close-contact criteria; (v) pre-positioned antibiotic and vaccine stockpiles; and (vi) mass communication infrastructure [32]. In the SSA context, these components should be considered applicable not only to tertiary campuses but also, where relevant, to secondary boarding-school environments that share comparable residential transmission risks. The UKHSA activated all six components in Kent, with documented limitations across each, as detailed in Section 3. Even in this benchmark response, the serogroup-typing component (iii) failed within the clinically relevant window.

### 7.2. The SSA Preparedness Deficit

Against this framework, SSA HEI preparedness is systematically deficient across all six components. Campus health clinics are typically staffed by general nurses and, at most, a general medical officer; parenteral ceftriaxone—first line for bacterial meningitis—is frequently unavailable at the campus level [16,30]. Preparedness protocols should also explicitly account for high-risk subpopulations, including students living with HIV and those with sickle cell disease, who may require tailored vaccination, prophylaxis, and clinical monitoring pathways. The relationship between antibiotic delay and meningococcal mortality is well established: Proulx et al. found that each additional hour of delay beyond the optimal window was associated with a statistically significant increase in mortality among adult bacterial meningitis cases [31], and the typical SSA campus-to-hospital transfer time of one to three hours represents a clinically critical deficit. Cerebrospinal fluid (CSF) examination and serogroup typing require referral to district or national reference laboratories, a process that takes days to weeks [26,27]. Contact tracing is impeded by the absence of student contact registries. Preparedness plans should also specify alternative prophylaxis regimens where local resistance patterns or national guidance limit reliance on ciprofloxacin as first-line post-exposure prophylaxis. Campus communication systems rely on unregulated, informal channels that are vulnerable to concurrent misinformation amplification [29].

### 7.3. Nightlife Transmission in the SSA HEI Context

The Kent outbreak’s nightclub linkage is directly transferable to SSA HEI cities, which host equally dense nightlife environments but face additional investigation challenges: absent formal attendance records, variable regulatory capacity for compliance, and limited public health contact-tracing authority over private venues [21,66]. Evidence from the single available SSA student study (Adegboye et al., noting its cross-sectional limitations) is consistent with nightlife attendance as a transmission amplifier in the Nigerian university context [66].

## 8. Recommendations: A Five-Pillar GRADE-Informed Framework

Drawing on the Kent 2026 outbreak, the SSA evidence base summarised in this review, and the WHO/Africa CDC preparedness guidance [32], we propose ten recommendations across five pillars: vaccination, surveillance, clinical-pharmaceutical preparedness, campus education, and governance. Each recommendation is graded for Quality of Evidence (QoE: High/Moderate/Low/Very Low) and Strength of Recommendation (SoR: Strong/Conditional) using GRADE-informed criteria [19]. Where strong recommendations are made despite moderate or low-quality evidence, this reflects the combination of biological plausibility, consistency with high-income country evidence, and the high-risk, time-sensitive nature of meningococcal outbreaks, where delayed action carries substantial consequences. Priority classification (HIGH 0–12 months; MEDIUM 12–24 months; LONG-TERM 2–5 years) reflects both the urgency of the evidence and the realistically achievable resource mobilisation timeline in most SSA settings. A Monitoring and Evaluation (M&E) Indicator is provided for each recommendation to support implementation tracking.

### Notes on Evidence Grading Rationale

Recommendations 1–4 (vaccination mandate, outbreak protocol, antibiotic pre-positioning, symptom training) receive Moderate or Low QoE because no randomised controlled trials or prospective cohort studies directly evaluate these interventions in SSA HEI settings; grading reflects extrapolation from the strong HIC campus evidence base and SSA general-population meningococcal evidence. Where QoE is Low, but SoR is Strong, this reflects that: the potential benefit is substantial; alternatives are inadequate; and the biological plausibility is well-established. Recommendation 9 (MenACWYX advocacy) receives High QoE because Phase II/III immunogenicity trial data directly support the vaccine’s effectiveness against the relevant SSA serogroups. Conditional recommendations (6, 7, 10) reflect genuine uncertainty about local implementation feasibility and resources.

## 9. Discussion

### 9.1. The Kent 2026 Outbreak as a Global Public Health Signal

The Kent outbreak provides a high-resolution case study of meningococcal transmission dynamics in a tertiary setting, offering a useful, though context-specific, reference point for considering preparedness in other regions: over 20 reported cases in the first week (including confirmed and suspected cases, later clarified as 21 laboratory-confirmed MenB cases) and 2 deaths within six days, spreading across two universities and multiple schools, despite the full force of UKHSA protocols, pre-existing vaccine programmes, and national pharmaceutical stockpiles. Described by the UK Health Secretary as “unprecedented” in pace and extent, the outbreak was confirmed as Neisseria meningitidis group B (MenB), not covered by the MenACWY vaccine routinely offered to UK teenagers, nor covered for the current university-age cohort by the infant MenB programme introduced in 2015. The speed of this event was not exceptional; it was precisely what the epidemiological literature predicts for an uncontained meningococcal cluster in a dense, social mixing environment [40,73]. Its significance lies in establishing a clear benchmark: if a sophisticated national public health system, coordinating 6500 antibiotic doses, a 5000-person MenB vaccination programme, and a national clinical alert across England within days, is still overwhelmed by a campus-origin meningococcal cluster that spread to a second university, the consequences of a comparable event on an SSA campus are sobering.

### 9.2. The Convergence of Risk in SSA Universities

The risk convergence in SSA universities is not merely analogous to the Canterbury outbreak; it is quantitatively greater across every measured dimension. The meningococcal threat environment is broader (five circulating serogroups versus one confirmed unprotected serogroup, MenB, in Canterbury); institutional defences are weaker across all six preparedness components; and structural amplifiers, such as residential overcrowding, co-infection with HIV and SCD, poor symptom literacy, and limited laboratory capacity, compound the baseline risk. The extension of the Canterbury outbreak to Canterbury Christ Church University (CCCU), likely driven by shared nightlife exposure, highlights how transmission networks in university cities may extend beyond single institutions. While this may reflect a common exposure event rather than sustained inter-institutional transmission, it nonetheless illustrates the potential for rapid multi-site involvement in dense urban student environments for SSA: in major African university cities, Accra, Lagos, Nairobi, Addis Ababa, Kampala, multiple universities and their students routinely share commercial nightlife spaces, creating multi-institutional cross-exposure networks of even greater density and complexity than Canterbury. A single campus cluster in an SSA city is, in structural epidemiological terms, simultaneously a multi-institutional risk event. No SSA city currently possesses any mechanism equivalent to the UKHSA contact-tracing infrastructure that identified and responded to the CCCU case within five days of the initial University of Kent cluster notification [11,16,32]. The limited published evidence based on SSA campus-specific meningococcal surveillance means that the true current burden remains uncertain, but the PHASA’s documentation of recurrent residential deaths at South African universities, normalised rather than systematically investigated, suggests that campus meningococcal mortality may already be occurring at a low but sustained rate across SSA [16].

Authors of this review acknowledge that extrapolating from HIC campus risk data to SSA necessarily entails limitations: different predominant serogroups, partially protective MenAfriVac coverage among current student cohorts, different co-infection profiles, and different healthcare-seeking norms. These differences may make the SSA risk both greater (through absence of any vaccination programme, greater residential density, diagnostic limitations) and potentially modified in ways not yet characterised (through partial MenAfriVac-acquired cross-immunity in some cohorts). Prospective SSA campus carriage and surveillance studies, recommended as a priority research action, are essential to replace this extrapolative framework with direct evidence.

### 9.3. The Cost-Effectiveness Dimension

The recommendation framework proposes interventions ranging from vaccination mandates to continental surveillance networks. The authors acknowledge that no direct cost-effectiveness analysis of university-entry meningococcal vaccination in SSA has been published. However, evidence from adjacent domains is informative: Colombini et al. documented that a single meningococcal epidemic in Burkina Faso generated household costs exceeding USD 100 per affected family—catastrophic in the local economic context—while community disruption, lost productivity, and healthcare system burden substantially multiplied the aggregate economic impact [28]. Published vaccine cost-effectiveness analyses for SSA, including for MenAfriVac and related programmes, consistently demonstrate favourable cost-per-DALY-averted ratios well below WHO cost-effectiveness thresholds [71]. The campus context, with its concentrated, mobile, economically productive student population, likely produces even more favourable cost-effectiveness outcomes than general-population vaccination, because the cost of a campus outbreak (hospitalisation, fatality, academic disruption, institutional reputational damage, legal liability) is concentrated and quantifiable. The assumption that university-entry vaccination is ‘affordable’ is contingent on Gavi-negotiated pricing for eligible countries; at USD 25–80 per dose in the private market, affordability requires public sector procurement and political commitment. This distinction must be central to any national policy advocacy using the recommendations in Table 1.

### 9.4. Regional Collaboration: Rationale, Precedents, and the SSA-MUAN

Section 3.1 documented that the Kent outbreak’s community transmission extended beyond the university to a secondary school, illustrating that meningococcal clusters do not respect institutional boundaries. In SSA, this challenge is amplified by transnational student flows: inter-African study mobility programmes now move tens of thousands of students across borders annually [13], meaning that a campus cluster originating in one country can have exposure chains extending across multiple national borders within 48 h of the index social event.

The existing WHO AFRO IDSR Alert and Response Operations (ARO) system provides a framework for cross-border disease alert sharing [55] but was designed for population-level surveillance and lacks the specificity and speed required for campus-cluster notification. The proposed SSA-MUAN draws design inspiration from two demonstrated precedents: the UKHSA’s campus notification infrastructure (the same infrastructure activated in Kent, enabling 30,000-person alerts within 48 h, archived procedures available at GOV.UK) [4], and the US American College Health Association (ACHA) meningococcal alert protocol, which enables real-time cross-institutional notification of confirmed campus meningococcal cases with serogroup and clinical data, facilitating coordinated prophylaxis recommendations across college health services nationally. No equivalent mechanism currently exists at any scale in SSA.

Three governance, financing, and data-sovereignty considerations must be addressed in any SSA-MUAN design. First, governance: the network should be hosted within the Africa CDC’s Integrated Disease Surveillance Platform [74] rather than as a standalone system, to leverage existing infrastructure and ensure integration with national disease notification pipelines. Africa CDC’s mandate explicitly encompasses pan-continental public health emergency response, making it the appropriate anchor institution. Second, data sovereignty: individual country participation should be governed by bilateral data-sharing agreements within the Africa CDC Information Sharing Agreements framework, with clear provisions on data ownership, privacy protection, and data-sharing limitations. Third, financing: initial establishment costs could be incorporated into the Global Fund’s post-2027 sub-Saharan Africa Health Systems Strengthening mechanism or World Bank Regional Disease Surveillance Systems Enhancement (REDISSE) programme, both of which already fund cross-border surveillance infrastructure; subsequent operational costs should be integrated into Africa CDC’s annual programme budget. A phased pilot, beginning with five to eight countries representing diverse belt, non-belt, and southern African epidemiological contexts, is recommended before continental rollout.

### 9.5. Strengths and Limitations

This narrative review, to the authors’ knowledge, is the first to systematically apply a HIC university meningococcal outbreak to SSA institutional health policy, drawing on a broad, cross-disciplinary evidence base with a GRADE-informed recommendation framework. Its principal limitation is the critical lack of direct SSA campus-specific meningococcal evidence, necessitating the tiered extrapolation approach described in Section 2.3. A second limitation concerns the temporal evolution of the outbreak data. The initial version of this review was drafted during the early phase of the Kent cluster (17–19 March 2026), when reported case counts included both confirmed and suspected cases. Subsequent UKHSA updates in early April 2026 clarified that the outbreak comprised 21 laboratory-confirmed MenB cases (18 outbreak strain) and two deaths. While these updates improve epidemiological precision, they do not materially alter the interpretation of the outbreak’s rapid progression or its policy implications. Our report’s substantive policy argument, resting on the structural vulnerabilities identified and the synthesised SSA evidence base, is not altered by these factual updates; the updated data, in fact, materially strengthen the argument by confirming that even within a well-resourced system, institutional containment within a single university failed. A third limitation is the potential for selection bias in study inclusion; a formal systematic review with quality-of-evidence grading per the GRADE approach would provide a stronger evidential foundation than is presented here. Fourth, publication bias may mean that campus meningococcal events in SSA managed without formal outbreak investigation are not reported in the literature, leading to a systematic underestimation of the true continental campus burden. This possibility strengthens rather than weakens the case for the campus surveillance investment recommended in Pillar 2. Use of the grey literature introduces potential variability in data completeness and verification; however, this is inherent to analyses of rapidly evolving outbreak events.

## 10. Conclusions

The 2026 Canterbury meningococcal outbreak, with two deaths, 20 confirmed and suspected cases within six days, and spread to two universities, establishes an important benchmark: even sophisticated public health systems are challenged by meningococcal cluster dynamics in university populations. The outbreak was confirmed as MenB, a serogroup not covered by the MenACWY vaccines routinely offered to UK teenagers, and not covered for the current university-age cohort by the 2015 infant programme. The extension to Canterbury Christ Church University via a shared nightlife exposure event confirms that single-institution containment strategies are insufficient in the absence of coordinated multi-institutional and community-level preparedness. For SSA, where the serogroup threat is broader (five circulating serogroups), the institutional defences are weaker across all six preparedness components, the structural risk amplifiers are greater, and the campus-specific evidence base is almost non-existent; the structural preconditions for a comparable or larger campus event appear to be present, although direct empirical confirmation in SSA tertiary settings remains limited.

The interventions most likely to reduce this risk are proportionate preparedness measures that combine earlier age-targeted vaccination in high-risk settings with university-level catch-up and campus protection measures, alongside antibiotic readiness, surveillance integration, symptom-recognition training, and regional alert mechanisms. Their implementation requires political will, institutional leadership, and coordination between the ministries of health and higher education, all achievable within existing policy frameworks.

The evidence reviewed here supports consideration of proactive preparedness measures in SSA tertiary settings, while highlighting the need for context-specific data to guide implementation. SSA universities, governments, and international health bodies should treat the Kent 2026 outbreak as a policy-motivating event and invest in the preparedness framework outlined in Table 1 before, rather than in response to, the first major SSA campus cluster.

## Figures and Tables

**Table 1:** GRADE-informed five-pillar recommendation framework for meningococcal preparedness in SSA universities.

Priority	Recommendation	QoE	SoR	Timeframe	Responsible Actor	M&E Indicator
HIGH	Implement meningococcal vaccination at key educational transition points in SSA, prioritising secondary-school entry in high-risk settings. Consider requiring documented meningococcal vaccination (MenACWY, where available; MenAfriVac in belt countries; MenACWYX when licenced) at key educational transition points, including university registration where feasible.	Moderate	Strong	0–12 months	Ministries of Higher Education; University Registrar Offices	% incoming students with documented meningococcal vaccination certificate at registration
HIGH	Develop and adopt a campus Meningococcal Outbreak Response Protocol (MORP) aligned with WHO IDSR frameworks, defining escalation thresholds, contact criteria, antibiotic pathways, communication structures, and designated public-health coordination contacts designated public-health coordination contacts.	Moderate	Strong	0–12 months	University Health Services; National Public Health Institutes	% SSA universities with a written, annually reviewed MORP by 2027
HIGH	Plan for pre-positioning campus-level stockpiles of parenteral ceftriaxone and locally appropriate prophylactic antibiotics, with pre-authorised outbreak dispensing protocols, documented resupply chains, and contingency plans aligned with national antimicrobial resistance patterns	Moderate	Strong	0–12 months	University Pharmacy Services; National Medicines Authorities	Number of campuses with verified prophylactic antibiotic stockpile meeting the defined minimum quantity
HIGH	Implement structured annual training in meningococcal symptom recognition and emergency response for relevant campus and residential personnel, including student leaders and, where applicable, linked boarding-school staff. Include HIV, SCD, gender, and subgroup-specific risk content. Training and communication strategies should explicitly target higher-risk subgroups, including male students, international students, and first-year entrants	Low	Strong	0–12 months	University Student Services; Campus Health; Student Unions	% target campus staff completing annual competency assessment with passing score
MEDIUM	Integrate campus health clinics into national IDSR passive surveillance systems and networks, with mandatory electronic notification of suspected invasive bacterial meningitis within 24 h and defined serogroup-typing referral pathways.	Moderate	Strong	12–24 months	WHO/AFRO; National Public Health Institutes; Ministries of Health; Africa CDC	Mean time from campus notification to national surveillance system entry, target <24 h
MEDIUM	Commission continent-wide meningococcal carriage studies in representative SSA HEI populations to characterise student serogroup distribution, carriage prevalence, and seasonal acquisition dynamics—data currently absent from the literature.	Very Low	Conditional	12–24 months	Academic Research Consortia; WHO/AFRO; Africa CDC; Gates Foundation	Number of peer-reviewed SSA campus meningococcal carriage studies published by 2028
MEDIUM	Engage nightlife venue operators in SSA HEI cities through meningococcal preparedness programmes, including symptom recognition materials, staff first-aid training, voluntary patron registration, and notification obligations upon patron illness reports.	Low	Conditional	12–24 months	Local Government Health Depts; Student Unions; Environmental Health Authorities	% nightlife venues in pilot university cities participating in preparedness programme
MEDIUM	Invest in a tiered diagnostic model for suspected campus clusters, combining faster decentralised presumptive diagnostic capacity where validated with strengthened regional serogroup-typing capacity, aiming for ≤72 h sample-to-result turnaround during outbreaks	Moderate	Strong	12–24 months	National Reference Labs; Africa CDC; NICD; Institut Pasteur Dakar	Mean turnaround time for serogroup confirmation during an outbreak event at enrolled labs
LONG-TERM	Advocate through the African Union Commission and the Gavi Alliance for accelerated MenACWYX regulatory approval, AMC eligibility, and programmatic rollout targeting university-entry cohorts across belt countries.	High	Strong	2–5 years	African Vaccine Regulatory Forum; Gavi; AU Commission; Serum Institute of India	Date of WHO pre-qualification and first-country EPI introduction of MenACWYX with student-cohort targeting
LONG-TERM	Establish a Sub-Saharan Africa Meningococcal University Alert Network (SSA-MUAN) modelled on the UKHSA–University of Kent campus notification framework and the US ACHA meningococcal alert protocol—a real-time, cross-border digital platform for campus cluster intelligence sharing among national public health institutes.	Low	Conditional	2–5 years	Africa CDC; WHO/AFRO; Association of African Universities; Ministries of Health	Number of SSA countries and universities enrolled in SSA-MUAN and receiving real-time alerts by 2030

QoE = Quality of Evidence (GRADE: High/Moderate/Low/Very Low). SoR = Strength of Recommendation (Strong/Conditional). M&E = Monitoring and Evaluation. Priority: HIGH = 0–12 months; MEDIUM = 12–24 months; LONG-TERM = 2–5 years.

## Data Availability

No new data was generated but all sources are cited in the reference list. Requests for additional methodological detail should be directed to the corresponding author.

## Supplementary Materials

The following supporting information can be downloaded at: https://www.mdpi.com/article/10.3390/idr18030051/s1, Supplementary File S1: SANRA Compliance Checklist. Supplementary File S2: Country-level SSA Meningococcal Vaccination Status Table. Supplementary File S3: Evidence Synthesis Summary Table—Key Studies on Campus Meningococcal Risk. Supplementary File S4: PRISMA-equivalent literature search flow diagram.
