# Supplementary material for: Meningococcal Outbreaks in Tertiary Education Settings in the United Kingdom: Lessons from the 2026 Kent Cluster for Surveillance, Vaccination Policy, and Institutional Preparedness in Sub-Saharan Africa—A Narrative Review
Source: Infect Dis Rep. 2026 May 26;18(3):51. doi: 10.3390/idr18030051 (PMC13299946; doi:10.3390/idr18030051)
Supplement: Supplementary file 1 [file idr-18-00051-s001.zip › idr-4239362-supplementary.pdf]

Supplementary Files

Supplementary File S1. SANRA Quality Assessment of the Narrative Review

The methodological quality of this narrative review was assessed using the SANRA checklist. The instrument comprises six items, each scored from 0 to 2 (0 = not addressed; 1 = partially addressed; 2 = fully addressed), with a maximum possible score of 12. Assessment was conducted by four reviewer(s), with discrepancies resolved through discussion.”

Table S1. SANRA compliance checklist

Criterion	Description	Score	Justification
1	Justification of the review's importance	2	The introduction (Section 1) clearly establishes the relevance of the review, referencing the 2026 Kent outbreak as a contemporaneous public health event and highlighting the limited availability of SSA campus-specific meningococcal data.
2	Statement of aims and literature search	2	Four explicit aims are defined (Section 1). The literature search strategy is detailed in Section 2.2, including databases, time frame (2000–2026), and keyword strategy.
3	Appropriate use of a database search	2	Multiple bibliographic databases (PubMed, Embase, and Scopus) and region-specific sources (African Journals Online) were searched. Grey literature sources (e.g., WHO, Africa CDC, UKHSA, CDC, Gavi) were also included, with supplementary hand-searching of reference lists.
4	Citation of appropriate literature	2	The review includes 82 references spanning peer-reviewed literature and institutional reports. Section 2.3 outlines a tiered evidentiary framework acknowledging heterogeneity and limitations in cross-context extrapolation.
5	Scientific reasoning	2	Interpretations are evidence-based, with explicit acknowledgement of limitations (Section 9.5). A GRADE-informed approach (Sections 2.4 and 8) is used to distinguish evidence quality from recommendation strength.
6	Appropriate presentation of data	2	Findings are presented using both tabular (e.g., Table 1) and narrative synthesis. The manuscript differentiates between primary data, secondary synthesis, and extrapolated findings (Sections 3–7).

Total SANRA Score: 12/12

Supplementary File S2. Country-Level Meningococcal Vaccination Status in Sub-Saharan Africa (March 2026)

This table summarises meningococcal vaccination policies across 46 sub-Saharan African (SSA) countries as of March 2026. Data were collated from World Health Organization vaccine-preventable disease monitoring systems (2025), Gavi, the Vaccine Alliance country programme documents (2023–2025), and Africa Centres for Disease Control and Prevention Expanded Programme on Immunisation (EPI) status reports (2024).

Table S2. Variables included in country-level dataset

Variable	Description
Country	WHO-recognised SSA country
WHO Region	WHO regional classification (AFRO)
Meningitis Belt Membership	Classification based on established African meningitis belt definitions
MenAfriVac in EPI	Inclusion of MenAfriVac (MenA conjugate vaccine) in national immunisation schedule
MenACWY Availability	Availability of quadrivalent conjugate vaccine (public, private, or unavailable)
University-entry Requirement	Presence of mandatory meningococcal vaccination for higher education entry
Notes	Recent outbreaks, policy updates, or relevant contextual factors

Summary Findings

- No SSA country had implemented an **HEI-entry meningococcal vaccination requirement** as of March 2026.
- MenAfriVac is included in routine immunisation schedules in **20 of 26 meningitis belt countries**.
- Quadrivalent MenACWY vaccines are **predominantly available through private-sector provision** (38/46 countries), with limited public-sector access.
- Reported pricing structures suggest **limited affordability for university student populations** in the absence of state-funded programmes.

Supplementary File S3. Evidence Synthesis of Key Studies on Meningococcal Risk in University Settings

This table presents a structured synthesis of studies informing meningococcal disease risk in higher education settings. Studies are categorised according to design, population, and evidentiary contribution, and are classified using the framework described in Section 2.3.

Table S3. Summary of included studies

Author (Year)	Setting	Study Design	Population	Key Finding	Limitations	Evidence Tier
Rosenstein et al. (1999)	USA	Observational	University students	Increased risk associated with dormitory residence	Older dataset; pre-conjugate vaccine era	High
Mbaeyi et al. (2019)	USA	Surveillance analysis	First-year students	11.8-fold higher MenB incidence among first-year students	Country-specific context	High
Soeters et al. (2019)	USA	Outbreak analysis	University campuses	Ten MenB outbreaks (2013–2018) in campus settings	Outbreak-driven dataset	High
Neal et al. (2000)	UK	Longitudinal carriage study	First-year students	Carriage increased from 6% to 23% during first year	Historical cohort	Moderate
Imrey et al. (2000)	USA	Cohort study	Dormitory residents	4.5-fold increase in carriage acquisition in dormitories	Limited generalisability	Moderate
PHASA (2025)	South Africa	Surveillance report	Residential students	Recurrent fatal cases in university residences	Grey literature; limited granularity	Moderate
Adegboye et al. (2021)	Nigeria	Cross-sectional	University students	Association between social behaviour and self-reported meningococcal illness	Self-report bias; cross-sectional design	Low

Abbreviation: PHASA = Public Health Association of South Africa

Interpretation:
The evidence base is dominated by high-income country outbreak and surveillance data, with **limited SSA-specific primary research**. The single SSA HEI-based analytical study is cross-sectional and hypothesis-generating.

Supplementary File S4. Literature Search and Study Selection Process (PRISMA-Adapted Flow Diagram)

The literature search and study selection process was conducted in accordance with transparency principles consistent with the PRISMA guidelines, adapted for narrative review methodology and aligned with the SANRA checklist (Criterion 3).

Identification

- Records identified through database searching:
    - PubMed/MEDLINE (n = 412)
    - Embase (n = 287)
    - Scopus (n = 341)

- Google Scholar (n = 593)
- African Journals Online (n = 88)

  Total database records: n = 1,721

- Additional records identified through grey literature sources (WHO, Africa CDC, UKHSA, CDC, Gavi): **n = 34**

Total records identified: n = 1,755

Screening

- Records after duplicate removal: n = 1,489
- Records screened (title and abstract): n = 1,489
- Records excluded: n = 1,327
    - Outside scope
    - Non-English/French
    - Pre-2000 (unless meeting predefined foundational criteria)

Eligibility

- Full-text articles assessed: n = 162
- Full-text articles excluded: n = 100
    - Non-meningococcal meningitis without comparative data (n = 31)
    - Paediatric-only populations (n = 28)
    - Insufficient primary data (n = 41)

Included

- Peer-reviewed studies included: n = 62
- Grey literature sources included: n = 20

Total sources included in narrative synthesis: n = 82

Reviewer Process

Study selection was conducted independently by two reviewers. Discrepancies were resolved through consensus discussion. No formal inter-rater reliability statistic was calculated, consistent with narrative review methodology.
